# Extracting Circadian and Sleep Parameters from Longitudinal Data in Schizophrenia for the Design of Pragmatic Light Interventions

**DOI:** 10.1093/schbul/sbab124

**Published:** 2021-11-10

**Authors:** Anne C Skeldon, Derk-Jan Dijk, Nicholas Meyer, Katharina Wulff

**Affiliations:** 1 Department of Mathematics, Faculty of Engineering and Physical Sciences, University of Surrey, GuildfordUK; 2 UK Dementia Research Institute Care Research & Technology Centre, at Imperial College London and the University of Surrey, Guildford,UK; 3 Surrey Sleep Research Centre, Faculty of Health and Medical Sciences, University of Surrey, Guildford,UK; 4 Department of Psychosis Studies, Institute of Psychology, Psychiatry and Neuroscience, King’s College London, >London, UK; 5 Insomnia and Behavioural Sleep Medicine Clinic, University College London Hospitals NHS Foundation Trust, London, UK; 6 Departments of Radiation Sciences and Molecular Biology, Umea University, Umea, Sweden; 7 Wallenberg Centre for Molecular Medicine (WCMM), Umea University, Umea, Sweden

**Keywords:** entrainment by light, transdiagnostic psychiatry, light therapy design, clinical predictive framework, model-data fusion, mathematical model

## Abstract

Sleep and circadian rhythm dysfunction is prevalent in schizophrenia, is associated with distress and poorer clinical status, yet remains an under-recognized therapeutic target. The development of new therapies requires the identification of the primary drivers of these abnormalities. Understanding of the regulation of sleep–wake timing is now sufficiently advanced for mathematical model-based analyses to identify the relative contribution of endogenous circadian processes, behavioral or environmental influences on sleep-wake disturbance and guide the development of personalized treatments. Here, we have elucidated factors underlying disturbed sleep-wake timing by applying a predictive mathematical model for the interaction of light and the circadian and homeostatic regulation of sleep to actigraphy, light, and melatonin profiles from 20 schizophrenia patients and 21 age-matched healthy unemployed controls, and designed interventions which restored sleep-circadian function. Compared to controls, those with schizophrenia slept longer, had more variable sleep timing, and received significantly fewer hours of bright light (light *>* 500 lux), which was associated with greater variance in sleep timing. Combining the model with the objective data revealed that non 24-h sleep could be best explained by reduced light exposure rather than differences in intrinsic circadian period. Modeling implied that late sleep offset and non 24-h sleep timing in schizophrenia can be normalized by changes in environmental light–dark profiles, without imposing major lifestyle changes. Aberrant timing and intensity of light exposure patterns are likely causal factors in sleep timing disturbances in schizophrenia. Implementing our new model-data framework in clinical practice could deliver personalized and acceptable light–dark interventions that normalize sleep-wake timing.

## Introduction

Disturbances in the duration, timing and quality of sleep are common in schizophrenia. Compared with controls, people with schizophrenia on average spend more time in bed, take more time to fall asleep, wake later and have more interrupted, fragmented sleep episodes.^1,2^ Sleep-wake patterns are more irregular over consecutive nights, and may be advanced, delayed or not synchronized with respect to the 24-h day.^1,3,4^

In schizophrenia, an expanding body of evidence has revealed associations between dysfunction of sleep and circadian timekeeping and greater severity in psychopathology,^5–7^ impaired cognition,^8,9^ and poorer quality of life.^10,11^ In the wider population, chronically disturbed and mistimed sleep are associated with obesity, cardiometabolic disease, physical inactivity and social withdrawal.^12,13^ These problems are over-represented in schizophrenia, and contribute significantly to premature mortality.^14^

Despite compelling arguments implicating sleep and circadian pathology in schizophrenia, interventions that target them have yet to become established in clinical practice. There is therefore a pressing need for acceptable, scalable, and cost-effective treatments that address sleep-circadian disturbance.

The design of effective interventions is facilitated by an understanding of the factors which give rise to the disorder. Two important variables that govern sleep-circadian timing have been identified in the general population. First, the intrinsic period of the circadian clock, ie, the duration of one cycle of the internal pacemaker, which is on average slightly longer than 24 h (24:09 h:m ± 0:12 h:m).^15^ Individuals with a period at the upper end of the population distribution are predisposed to misaligned sleep.^16^ Second, the light exposure pattern, which is the primary stimulus through which the circadian clock and therefore sleep timing is synchronized to the 24-h day. Insufficient or mistimed exposure to light results in variable sleep that is misaligned to the 24-h day in sighted^17^ and blind individuals.^18^ In schizophrenia however, the relative contributions to sleep-circadian dysregulation arising from endogenous biological factors such as the intrinsic circadian period on the one hand, and influences related to the light environment, psychopathology or medication effects, on the other, are unclear.

A powerful approach to disentangling causal influences is to combine longitudinal data with predictive physiologically informed dynamic mathematical models. These models are now so effective that they can explain circadian timing in a range of conditions,^19,20^ and have been used to gain insights into the mechanisms underlying the effects of age, light and social constraints on sleep.^21–23^

Here, we use a physiologically informed model to quantitatively analyze sleep–wake patterns using sleep diary and light data from community-dwelling patients with schizophrenia and unemployed controls. By extracting individual model parameters, we examine the relative contribution of physiological and environmental factors and then suggest environmental light exposure patterns to regularize sleep. We propose a pathway for implementation in clinical practice.

## Materials and Methods

### Data source and Protocol

Data were collected over a period of 6 weeks from 20 individuals meeting the DSM-IV criteria for a diagnosis of schizophrenia (mean age (SD) 38.8 (8.6) years, 5 women), and 21 healthy unemployed age-matched controls (within 5 years, mean age (SD) 37.5 (9.6) years, 8 women) from London. All patients were receiving medication and in a clinically stable state.

The study protocol was conducted in accordance with the Declaration of Helsinki and approved by the West London Mental Health Trust Local Research Ethics Committee and, for one of the participants, by the Lothian Local Research Ethics Committee. All participants gave written informed consent.

Light exposure patterns, actigraphy, melatonin profiles and sleep diary data were used in the current analyses. The feasibility and acceptability of the data collection methods^24^ and a descriptive analysis of sleep patterns^1^ have been previously reported. All participants from^1^ were included in the current analyses. Below we briefly summarize the data but further details are available in the Supplementary Material.

### Monitoring of Sleep–wake Timing and Light Exposure

Objective rest-activity and light profiles were sampled continuously in 2-min epochs over 6 consecutive weeks using the Actiwatch-L (Cambridge Neurotechnology Ltd, Cambridge, UK). In addition, all participants kept daily records of bedtime, get-up time, daytime activities, and deviations from their habitual routines such as celebrations. Assessments of “bedtime” and “get-up time” were inserted into the “Actiwatch Activity and Sleep Analysis” software (CamNtech UK) for automated calculation of “sleep onset” and “sleep offset” times.

### Analysis of Sleep–Wake Cycles and Light Exposure

**Fig. 1.**
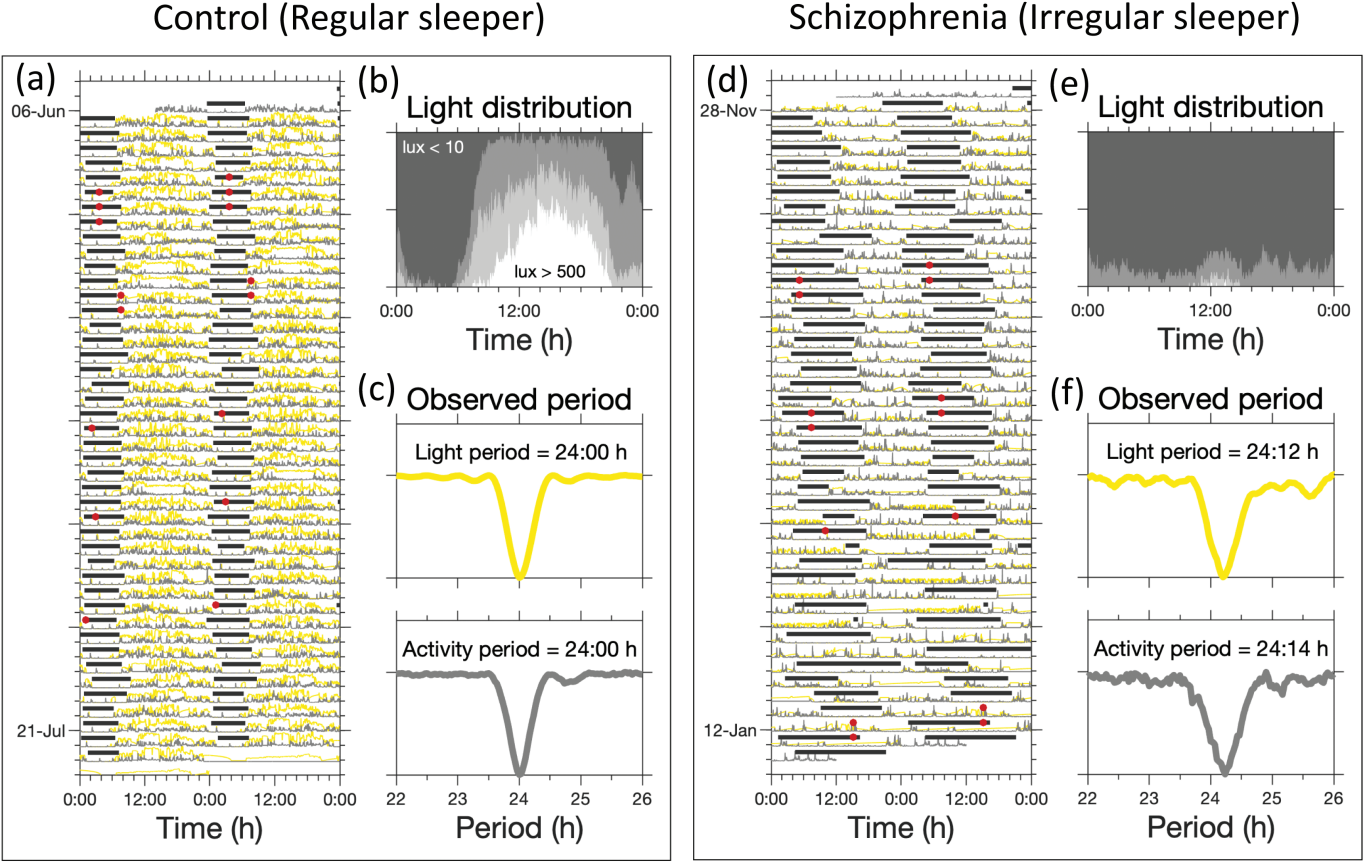
Typical rhythms of sleep, activity and light exposure. (a), (d): Light (yellow trace), activity (grey trace), 6-sulphatoxymelatonin (aMT6s) acrophase (red circles) and sleep timing (horizontal grey bars). (b) and (e): Average pattern of light exposure across the 24-h day. The shaded regions from white through to dark grey indicate the fraction of time spent at different light levels (*>*500 lux; between 500 and 100 lux; between 100 and 10 lux, *<*10 lux). (c) and (f) Residual variance as a function of period for activity and light. These indicated that light and activity followed a 24 h pattern for the regular sleeper, but a longer than 24-h pattern for the irregular sleeper.

The periodicities of the rest-activity and light–dark cycle were assessed by a non parametric approach which makes no assumptions about the waveform of these rhythms, figure 1. In this approach, data were folded at a range of periodicities in steps of 2 min, ie, the resolution of the actigraphy data. The period which resulted in the smallest residual variance was considered the dominant period.^25^ The procedure was implemented in MATLAB.^26^

Sleep duration was calculated as the time from sleep onset to sleep offset, with mid sleep timing being the midpoint of each sleep episode. Mean and variance of sleep duration were calculated using linear statistics. Since some participants exhibited very large day-to-day differences in sleep timing, individual mean and variance of sleep onset, sleep offset and mid sleep were calculated using circular statistics.^27^

For each participant, three measures that quantified their light exposure were calculated. These were (i) the median number of hours of bright light per day (light intensity *>* 500 lux), (ii) the median daily light intensity (lux), (iii) the median daily light intensity on a log scale (log(lux + 1)).

### Assessment of Circadian Phase From Melatonin Measurements

Over a 48-h period once a week, 17 out of 20 people with schizophrenia and all 21 people in the control group completed repeated urine collection for determination of circadian acrophase for 6-sulphatoxymelatonin (aMT6s) concentrations.^28,29^

### Estimating Determinants of Sleep Timing by Model-fitting

For each individual, we fed the raw light data into a nonlinear differential equation model that captures the known core physiological processes underlying sleep–wake regulation including sleep homeostasis and circadian rhythmicity^30,31^ and the effects of light exposure on the circadian clock.^21,23,32^

In order to capture individual differences in physiological sleep need and circadian regulation, we fitted two essential parameters of the model namely the mean drive for wakefulness and the intrinsic circadian period.

### Using the Mathematical Model to Design Light Interventions

The intensity, color, duration and timing of light could all be tuned, leading to many different possible light interventions. Our aim was to develop approaches to promote earlier wake time and synchrony with the 24-h day, that were low-burden. We therefore focussed on two particular dimensions: increasing the overall amount of light available during daytime hours and/or decreasing the overall amount of light available during the evening/night.

To demonstrate the impact of daytime versus evening light, we constructed an average 24-h profile of “available” light that smoothly climbs to a maximum value at around midday and then decreases to a fixed level during the evening/night.^23^ Within the model, this available light is turned on during model-predicted wake, and is turned off during model-predicted sleep. Thus the model is not forced to wake up, but self-selects when to be exposed to light. This self-selection of light is critical for quantitative prediction of circadian phase and sleep timing.^33^

Further extensive modeling details are given in the Supplementary Material.

### Statistics

Key variables (variance of sleep onset and offset, mean daily hours of bright light, fitted intrinsic circadian period, fitted sleep drive) for schizophrenia and control participants were compared using the Wilcoxon rank-sum test. Correlations that factored out the group effect were tested using the non parametric Spearman’s rho coefficient using MATLAB’s partialcorr function. For the repeated measures of sleep duration, daily sleep onset and offset a linear mixed-effect modeling analysis was carried out.

Data from one participant with schizophrenia were collected twice, in the summer and again in the winter. Since measures of light and of sleep timing differed substantially between the two seasons this participant was included twice in all analyses of light and sleep timing measures but fitting was only carried out in one season (summer).

## Results

### Lower Levels of Bright Light Exposure were Associated with Greater Variance in Sleep Timing

Rest-activity, light exposure, sleep timing, and melatonin phase recorded simultaneously and longitudinally showed that, relative to controls, those with schizophrenia were heterogenous in their sleep–wake patterns, exhibiting excessively long, irregular or fragmented sleep and misalignment with the 24-h day.^1^ Examples from control and schizophrenia participants are shown in figure 1(a) and (d).

**Fig. 2.**
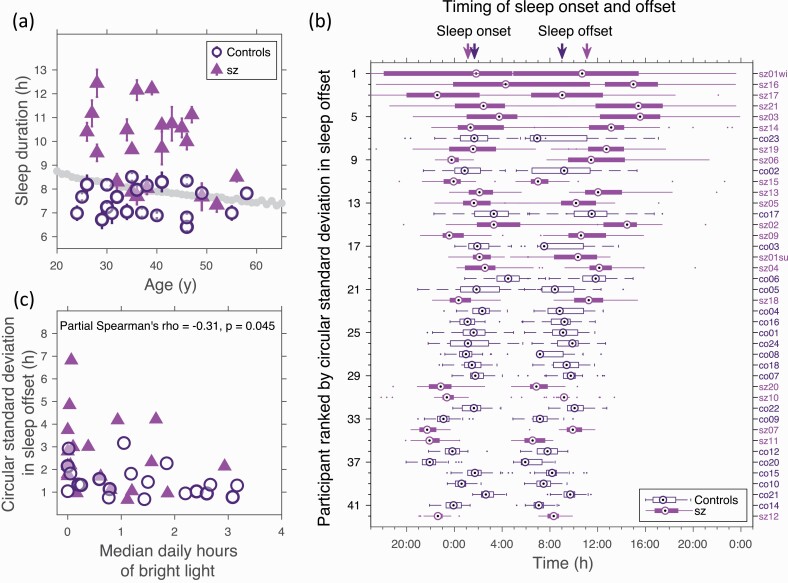
Sleep is longer and more irregular in people living with schizophrenia than in healthy unemployed controls with irregular sleep associated with fewer hours of bright light (a) Sleep duration as a function of age for both groups. The grey line is the population average from a large self-report survey.^48^ (b) Box and whisker plots showing sleep onset and offset timing for each participant. Each “box” goes from the 25th to the 75th percentiles of sleep onset/offset. The length of the whiskers is an indicator of the range. The circles within each box give the median value. Participants ranked 1,2,4 and 5 were classified in ^1^ as having non 24-h rhythms. The arrows mark mean sleep onset and offset times for each group. (c) Standard deviation in participant sleep offset versus participant median daily hours of bright light (light *>*500 lux). Data were collected from participant sz01 in both summer and winter. Since this individual exhibited large seasonal differences in light exposure and sleep timing, in panel (b) and (c) they appear twice (sz01su and sz01wi for summer and winter respectively).

Specifically, the average sleep duration was 2:24 h:m longer in those with schizophrenia than in controls, (*p <* 0.0001), see figure 2(a). Sleep onset in those with schizophrenia was similar to controls (0:30 h:m earlier, *p* = 0.36), but sleep offset was significantly later (2:07 h:m later, *p* = 0.0004), see supplementary table S2.

The variance of both sleep onset and offset times in those with schizophrenia was significantly greater than in the unemployed controls (*p* = 0.007 and *p* = 0.010, respectively, *n* = 21 for both groups), figure 2(b). The two participants of figure 1 are close to the extremes of variance in sleep offset ranking 5th and 39th out of 42, respectively.

Many participants were exposed to little bright light, with more than half the participants (55%, 14 sz, 9 controls) receiving less than one hour of bright light (*>* 500 lux) on a typical day. The two examples shown in figure 1(b) and (e) are close to the extremes, and were exposed to daily medians of 3:06 h:m and no hours of bright light, respectively. Furthermore, the variation in sleep offset was significantly correlated with the median daily number of hours of bright light exposure, figure 2(c), the mean daily light exposure, (*p* = 0.030, *n* = 21 in each group) and the mean daily log light exposure, (*p* = 0.043, *n* = 21 in each group).

### Combining Light Exposure Data with a Personalized Mathematical Model Captured Average Sleep Timing and Duration

**Fig. 3.**
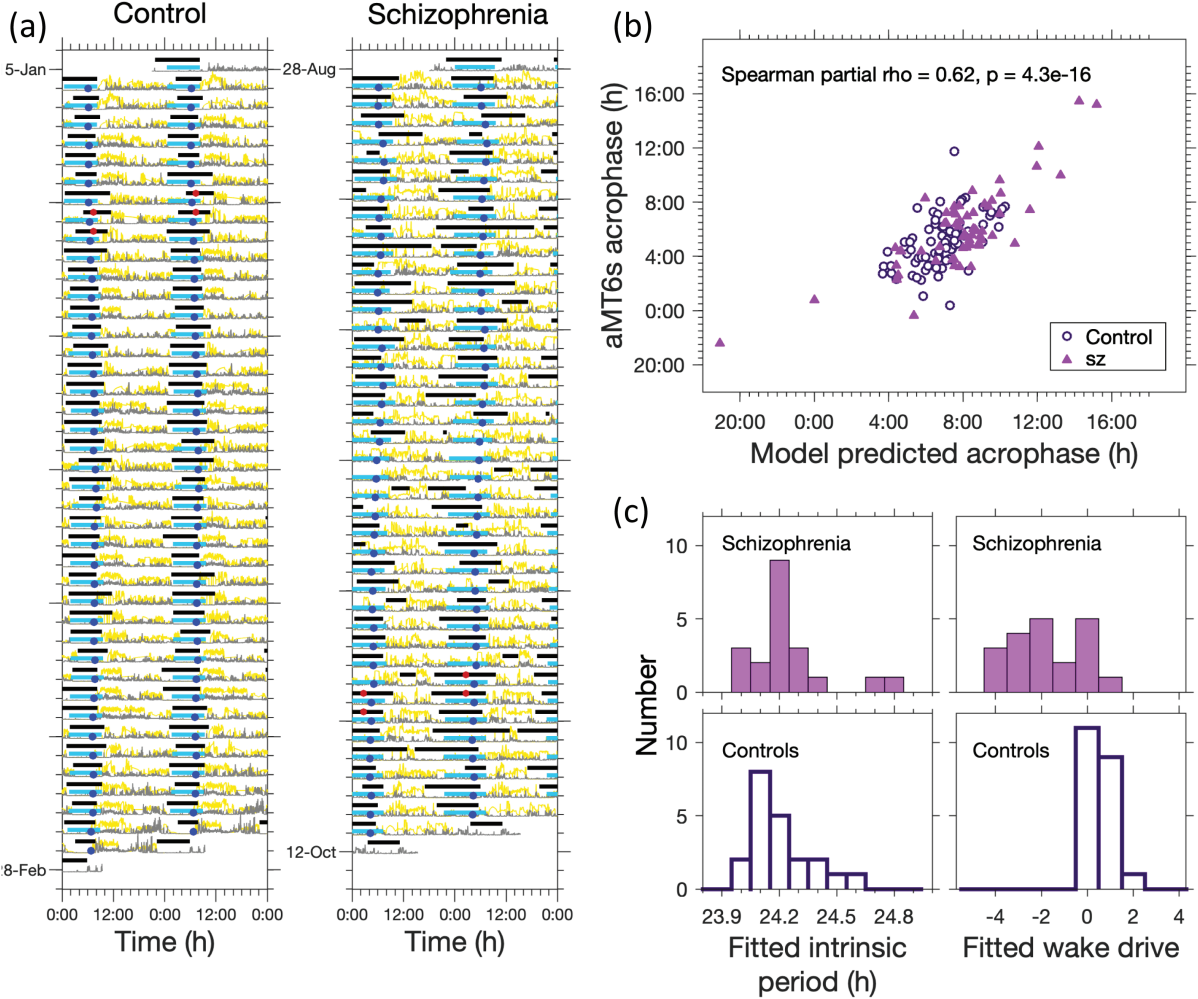
Combining light data with a personalized mathematical model for sleep and circadian rhythms explains sleep duration and mean sleep timing in both those with schizophrenia and healthy controls. (a) Two example fits of the model for a regular sleeper (control) and an irregular sleeper (schizophrenia). Observed light exposure (yellow trace), activity (grey trace), 6-sulphatoxymelatonin (aMT6s) acrophase (red circles) and diary sleep timing (horizontal grey bars), are shown. The horizontal light blue bars show model predicted sleep timing with model predicted circadian wake propensity minima as blue circles. (b) aMT6s acrophase measurements versus model predicted acrophase. (c) Distributions of fitted model parameters (intrinsic circadian period and wake drive) for those with schizophrenia and controls.

Fitting the mean wake drive and the intrinsic circadian period in the mathematical model enabled mean sleep duration and timing to be matched for every individual, as shown for two examples in Figure 3(a), one for a control participant with regular sleep, and one for a schizophrenia participant with variable day-to-day sleep. The estimated intrinsic periods were 24:10 h:m and 24:10 h:m and the estimated wake drives were 0.46 and −1.73 (deviation from default value), respectively.

As further validation of our modeling approach, for each participant, we compared each urinary aMT6s acrophase measurement with the corresponding model predicted circadian wake propensity minimum. There was a strong correlation between modeled and observed acrophase (figure 3(b) and supplementary table S2).

The fitted values of intrinsic circadian period all fell within a physiologically plausible range (mean (SD) 24:13 (0:11) h:m; range (23:58, 24:50) h), as observed in protocols specifically designed to assess intrinsic period.^15^ The distributions for fitted intrinsic circadian period did not differ significantly between people living with schizophrenia and controls (sz: mean (SD) 24:14 (0:12) h:m; Controls: 24:13 (0:10) h:m; *p* = 0.67) figure 3(c).

Conversely, the distribution of the fitted mean wake drive in schizophrenia was significantly different from that for controls (sz: mean (SD) −1.82 (1.62); Controls: 0.66 (0.62); *p<* 0.0001), figure 3(c), suggesting that the longer sleep durations in schizophrenia may be explained by a reduced drive for wakefulness.

### Designing Pragmatic, User-friendly Light Interventions to Normalize Sleep Timing and Synchrony Using a Mathematical Model

Since the model was able to quantitatively predict sleep duration and timing using raw light data, we next simulated a range of different “interventions” to normalize circadian rhythmicity. For illustrative purposes, we used the light exposure profile of the participant ranked 1st in figure 2(b) who exhibited non 24-h rhythms as a starting point, see supplementary figure S3.

**Fig. 4.**
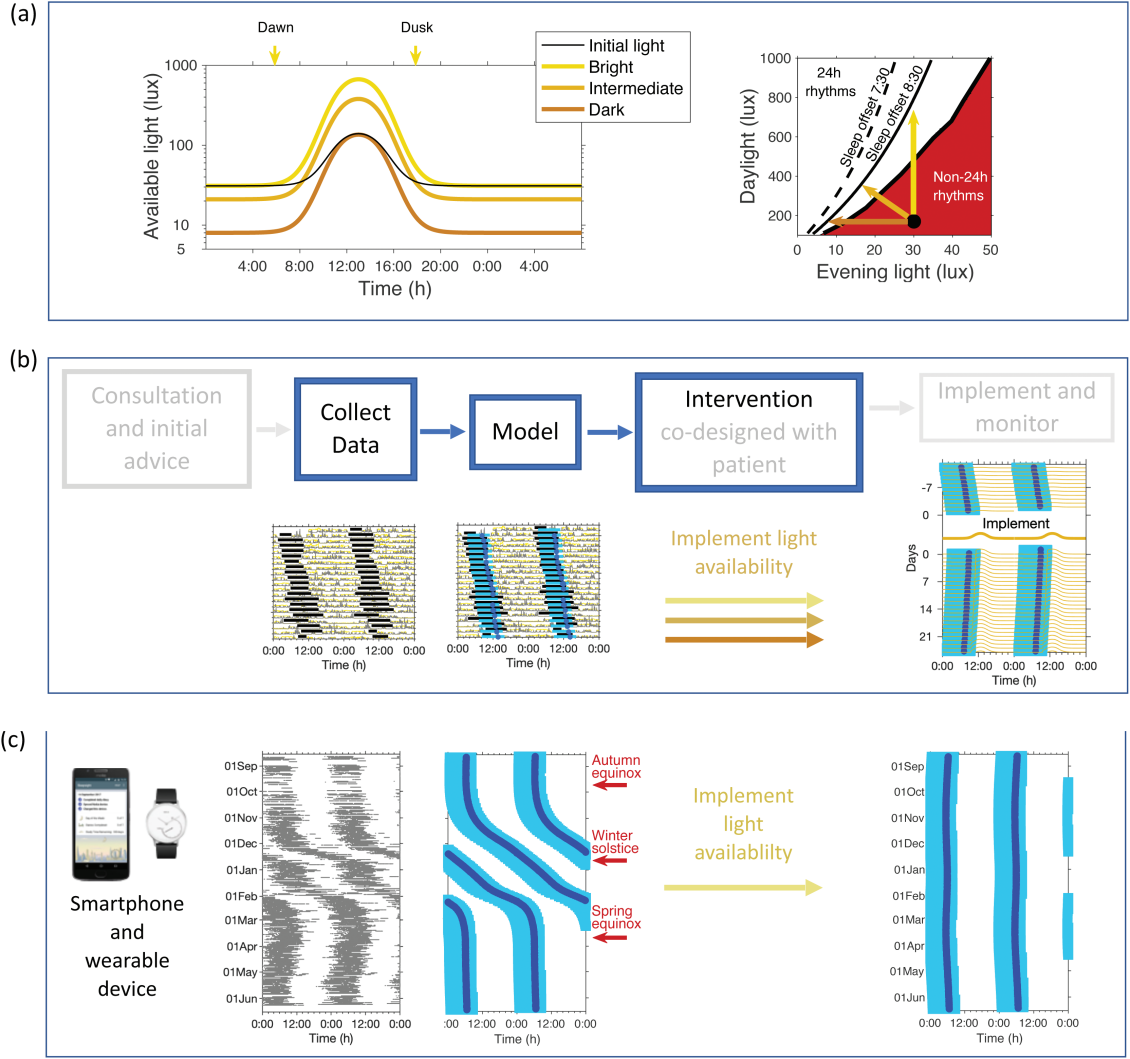
Design of personalized light interventions and their incorporation in a clinical implementation framework. (a) Lefthand panel: the black line shows the average light distribution for participant sz01 in the winter when they exhibited non 24-h rhythms. The three colored lines indicate three different available light exposure profiles which would result in 24 h rhythmicity with a wake time of 08:30. The righthand panel shows the region of 24 h and non 24-h rhythms as a function of the intensity of the daylight versus the intensity of light in the evening. The black dot indicates the position of the initial light profile, with the three arrows indicating the three different light interventions. (b) Suggested clinical implementation. (c) Future personalized data collection, intervention and monitoring using smart technology, here illustrated as an extension to the Sleepsight project.^34^ The rest-activity pattern observed in one participant in the Sleepsight project is shown to the left. The modeled sleep-timing is shown in the middle, with the predicted sleep from an individually designed light exposure profile to the right. All panels: periods of observed rest are shown in grey. Modeled sleep timing is shown in blue with the dark blue line showing the circadian wake propensity minima.

Three alternatives are shown in figure 4(a), left panel. These are (i) increasing the peak environmental or available light level during the day from 160 to 795 lux; decreasing the amount of available light in the evening from 30 to 7 lux; or a mixture of the two, with a moderate increase in available daytime light (from 160 to 450 lux) and a moderate decrease in available evening light (from 30 to 20 lux). Each of these profiles has the same result of restoring a 24 h rhythm with a mean sleep offset of 08:30 in someone with a non 24 h rhythm. The finding that *different* light interventions result in the *same* sleep timing is further highlighted in figure 4(a) (righthand panel) where the results of approximately 4500 simulations are shown. figure 4(a) illustrates that access to light of insufficient intensity during the daytime or of too high an intensity in the evening result in non 24-h rhythms. The precise position of the boundary that separates 24-h from non 24-h rhythms and the lines indicating sleep offset times, are dependent on individual physiological characteristics, see supplementary figure S4. For example, a longer intrinsic period means that a “stronger” signal is needed for entrainment.

Figure 4(b) proposes an intervention framework for the application of personalized light interventions in the clinic which could be incorporated in a remote monitoring platform, for example Sleepsight^34^ as outlined in figure 4(c). In this framework, initial consultation and advice is followed by quantifying sleep–wake and light exposure patterns, for example using wearable devices. With an appropriate software interface, an intervention consisting of a light-availability profile could then be co-designed with an individual taking into account individual physiological characteristics and personal preferences.

### Altered Light Profiles were Sufficient to Regularize Sleep Timing and Synchrony: A Natural Experiment

**Fig. 5.**
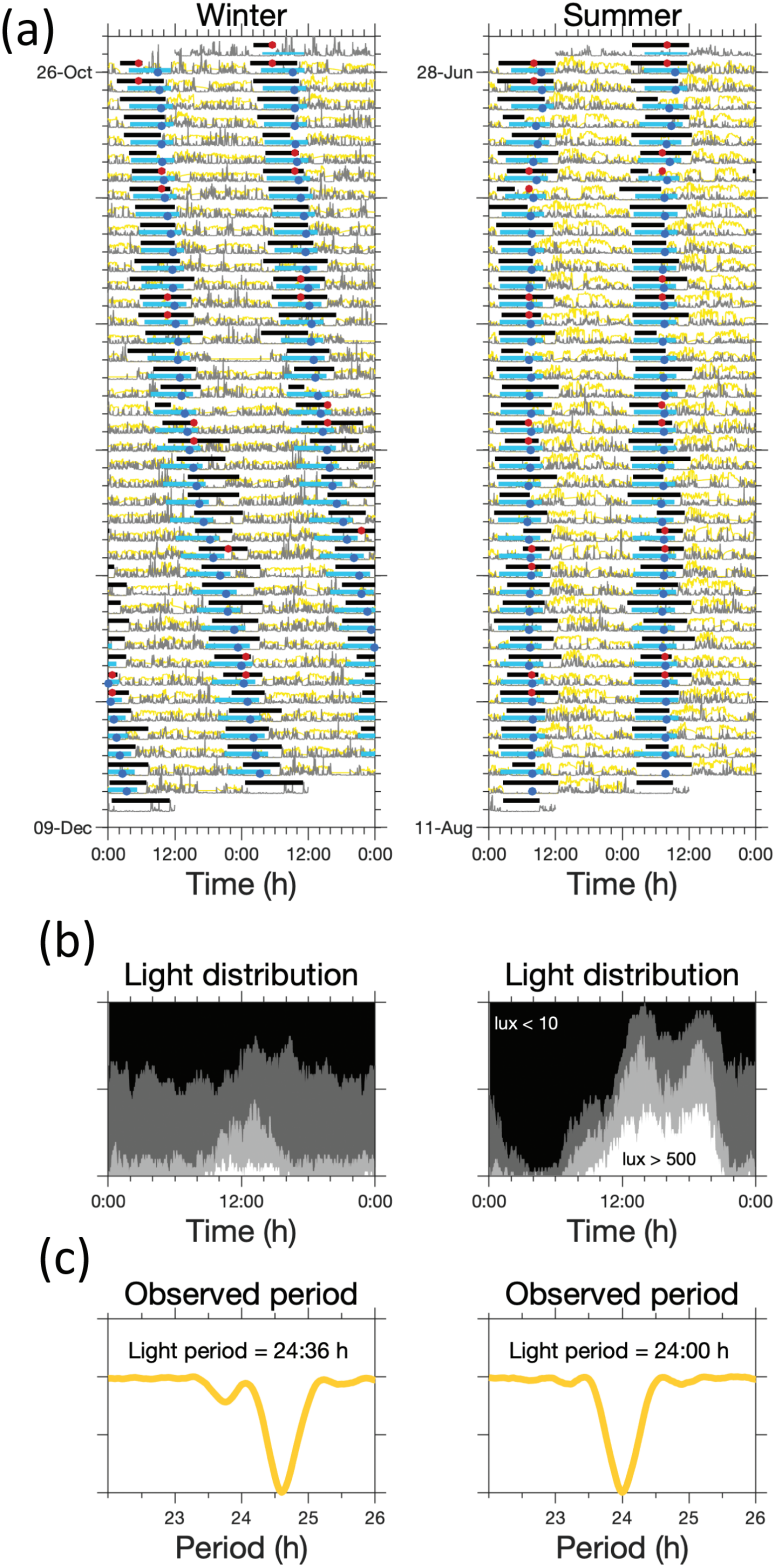
Natural experiment: changes in light exposure patterns can regularize sleep timing. (a) Observed light (yellow lines), activity (grey lines), diary sleep timing (horizontal grey bars) and 6-sulphatoxymelatonin (aMT6s) acrophase (red circles), in winter and in summer respectively. The horizontal light blue bars show model predicted sleep timing with model predicted circadian wake propensity minima shown by the blue circles. (b) Average patterns of light across the 24 h day in the winter and summer respectively. The shades of the regions from white through to dark grey indicate the fraction of time spent at different light levels (*>*500 lux; between 500 and 100 lux; between 100 and 10 lux, *<*10 lux). (c) The residual variance as a function of period for light, further highlighting the non 24-h rhythm in the winter that is regularized by the brighter light in the summer.

Light, activity and diary data were collected from one participant during the summer and again during the winter, see figure 5. A regular pattern of sleep was observed during summer but a non 24-h rhythm in winter (figure 5, summer: activity, light, melatonin periods are all 24:00 h:m. Winter: periods are 24:38 h:m, 24:36 h:m and 24:34 h:m, respectively). Consistent with seasonal changes this participant was exposed to less bright light in winter and for a shorter duration.

The output of the mathematical model accurately replicated the differences in synchrony between winter and summer. In the simulation, the only differences between seasons were the light data fed into the model, arguing for reduced light exposure being a parsimonious explanation for the emergence of non 24-h rhythms for this participant.

## Discussion

This study confirms previous findings of significantly increased sleep duration and more variable sleep timing in schizophrenia.^2^ Greater variability in sleep timing is associated with lower subjective sleep quality, as well as poorer mental and physical health outcomes.^35,36^

Our predictive modeling approach, adds to this by demonstrating that a combination of significantly reduced drive for wakefulness, together with reduced light exposure, simulated the longer sleep duration and later sleep offset seen in schizophrenia precisely and parsimoniously.

Our findings argue against the existence of core abnormalities in the circadian apparatus, and suggest that the disturbances observed in schizophrenia arise as a consequence of insufficient light exposure. A reduced circadian drive for wakefulness is likely to be influenced by the effects of sedative medication, the sleep and waking environment, co-morbid sleep disorder, and psychopathology including the negative symptom dimension, anxiety and low mood,.^37,38^

Foremost, our results underscore how light exposure is a powerful cue for synchronizing the internal clock with the day–night cycle,^39^ reducing sleep drive and increasing alertness. We therefore suggest that existing cognitive behavior therapies for insomnia (CBT-I) be adapted for this population, by emphasizing pragmatic circadian interventions that do not necessarily require technologies such as timed bright light therapy from light boxes, which are time consuming and not easily embedded into daily routines.^40^ Increasing light exposure can be facilitated by simple and inexpensive behavioral interventions, such as sleep and activity schedules which encourage regular exposure to daylight and modifications that promote natural light in living spaces. Providing lux meters may also help.

We have also demonstrated how light exposure does not require precise timing. Light exposure is determined by the light available and the behavior of the participant. Our simulations indicate that stable entrainment is achieved by consistent light exposure of sufficient intensity during daytime hours, and low enough intensity in the evening. The rationale for our approach is based on well-established theoretical principles and empirical observations in many species, including humans.^41^ We emphasize, that unlike traditional approaches,^42^ our approach does not require participants to wake early, but instead suggests a desirable light exposure profile which is “sampled” by participants according to their sleep schedule.

Second, it is necessary to concurrently address factors that reduce wake drive. Achieving minimum effective doses of sedative antipsychotic medications, together with careful consideration of the timing of both sedative and activating medications, is warranted. Tackling sleep hygiene problems by minimizing time spent awake in bed, separating the sleep and wake environments, and scheduling regular daytime activity, are key to mitigating against behaviors which diminish wake propensity. The direct, non circadian, positive effects of light on alertness will also improve sleep-timing.^43^ Addressing factors interfering with sleep such as psychotic symptoms, nightmares, racing thoughts or dysfunctional thoughts relating to sleep is also important.

Finally, differences in physiology and personal preference mean that what works for one person may not work for another. We therefore propose a clinical intervention framework that combines digital technologies for light and sleep–wake monitoring with model-data fusion to design bespoke therapies that take into account individual physiological characteristics (Fig. 4(b)). Furthermore, since different light exposure profiles can lead to the same outcome, co-design allows the patient to select a light availability profile that best suits their schedules, thereby facilitating adherence.

Our study has several strengths and limitations. As strengths, we point to the novel modeling approach which combines multi-variable, real-world longitudinal data with established models of sleep–wake timing. We note that the quantitative nature of the model means that the effect of *a*ny light profile can be predicted. Our method may be generalizable to other disorders, for example those with unipolar depression, bipolar disorder or dementia.^44^ However, we also recognize that modeling between-day variability in sleep timing, for example as in,^45^ and short-term alerting effects of light is pending. Further data are also required to examine the extent to which increasing light alters sleep duration. Our sample contained more men than women, although we found little evidence for sex differences in sleep measures or fitted parameters (see Supplementary Material). Abnormalities in sleep architecture have been identified in schizophrenia,^46^ and further work is required to examine the important interaction between the circadian system and sleep architecture^47^ in this population.

## Supplementary Material

sbab124_suppl_Supplementary_Material

## Data Availability

Code is available from the first author on reasonable request. Sharing of specific datasets is by reasonable request to the senior author, who collected and holds the data.

## References

[1] Wulff K , Dijk DJ, Middleton B, Foster RG, Joyce EM. Sleep and circadian rhythm disruption in schizophrenia. Br J Psychiatry. 2012;200(4):308–316. 22194182 10.1192/bjp.bp.111.096321 PMC3317037

[2] Meyer N, Faulkner SM, McCutcheon RA, Pillinger T, Dijk D-J, MacCabe JH. Sleep and circadian rhythm disturbance in remitted schizophrenia and bipolar disorder: a systematic review and meta-analysis. Schizophrenia Bull. 2020;46:1126–1143. 10.1093/schbul/sbaa024 PMC7505194 32154882

[3] Wirz-Justice A , Haug HJ, Cajochen C. Disturbed circadian rest-activity cycles in schizophrenia patients: an effect of drugs? Schizophr Bull. 2001;27(3):497–502. 11596850 10.1093/oxfordjournals.schbul.a006890

[4] Kodaka M , Tanaka S, Takahara M, et al. Misalignments of rest–activity rhythms in inpatients with schizophrenia. Psychiat Clin Neuros. 2010;64:88–94. 10.1111/j.1440-1819.2009.02047.x 20015119

[5] Afonso P , Brissos S, Figueira ML, Paiva T. Schizophrenia patients with predominantly positive symptoms have more disturbed sleep-wake cycles measured by actigraphy. Psychiatry Res. 2011;189(1):62–66. 21257208 10.1016/j.psychres.2010.12.031

[6] Mulligan LD , Haddock G, Emsley R, Neil ST, Kyle SD. High resolution examination of the role of sleep disturbance in predicting functioning and psychotic symptoms in schizophrenia: a novel experience sampling study. J Abnorm Psychol. 2016;125(6):788–797. 27362488 10.1037/abn0000180

[7] Kasanova Z , Hajduk M, Thewissen V, Myin-Germeys I. Temporal associations´ between sleep quality and paranoia across the paranoia continuum: an experience sampling study. J Abnorm Psychol. 2020;129:122–130. 31343182 10.1037/abn0000453

[8] Martin J , Jeste DV, Caliguiri MP, Patterson T, Heaton R, Ancoli-Israel S. Actigraphic estimates of circadian rhythms and sleep/wake in older schizophrenia patients. Schizophr Res. 2001;47(1):77–86. 11163547 10.1016/s0920-9964(00)00029-3 PMC2758687

[9] Bromundt V , Koster M, Georgiev-Kill, et al. Sleep–wake cycles and cognitive functioning in schizophrenia. Br J Psychiat. 2011;198:269–276. 10.1192/bjp.bp.110.078022 21263013

[10] Hofstetter JR , Lysaker PH, Mayeda AR. Quality of sleep in patients with schizophrenia is associated with quality of life and coping. BMC Psychiatry. 2005;5:13. 15743538 10.1186/1471-244X-5-13 PMC554780

[11] Reeve S , Sheaves B, Freeman D. Sleep disorders in early psychosis: incidence, severity, and association with clinical symptoms. Schizophr Bull. 2019;45(2):287–295. 30202909 10.1093/schbul/sby129 PMC6403049

[12] Schmid SM , Hallschmid M, Schultes B. The metabolic burden of sleep loss. Lancet Diabetes Endocrinol. 2015;3(1):52–62. 24731536 10.1016/S2213-8587(14)70012-9

[13] Roenneberg T , Merrow M. The circadian clock and human health. Curr Biol. 2016;26(10):R432–R443. 27218855 10.1016/j.cub.2016.04.011

[14] Laursen TM , Nordentoft M, Mortensen PB. Excess early mortality in schizophrenia. Annu Rev Clin Psychol. 2014;10:425–448. 24313570 10.1146/annurev-clinpsy-032813-153657

[15] Duffy JF , Cain SW, Chang AM, et al. Sex difference in the near-24-hour intrinsic period of the human circadian timing system. Proc Natl Acad Sci U S A. 2011;108 Suppl 3:15602–15608. 21536890 10.1073/pnas.1010666108 PMC3176605

[16] Micic G , Lovato N, Gradisar M, Burgess HJ, Ferguson SA, Lack L. Circadian melatonin and temperature taus in delayed sleep-wake phase disorder and non-24-hour sleep-wake rhythm disorder patients: an ultradian constant routine study. J Biol Rhythms. 2016;31(4):387–405. 27312974 10.1177/0748730416650069

[17] Duffy JF , Wright KP Jr. Entrainment of the human circadian system by light. J Biol Rhythms. 2005;20(4):326–338. 16077152 10.1177/0748730405277983

[18] Lockley SW , Arendt J, Skene DJ. Visual impairment and circadian rhythm disorders. Dialogues Clin Neurosci. 2007;9(3):301–314. 17969867 10.31887/DCNS.2007.9.3/slockley PMC3202494

[19] Stone JE , Postnova S, Sletten TL, Rajaratnam SMW, Phillips AJK. Computational approaches for individual circadian phase prediction in field settings. Curr Opin Sys Biol. 2020;22:39–51.

[20] Dijk DJ , Duffy JF. Novel Approaches for assessing circadian rhythmicity in humans: a review. J Biol Rhythms. 2020;35(5):421–438. 32700634 10.1177/0748730420940483 PMC7543025

[21] Phillips AJ , Chen PY, Robinson PA. Probing the mechanisms of chronotype using quantitative modeling. J Biol Rhythms. 2010;25(3):217–227. 20484693 10.1177/0748730410369208

[22] Skeldon AC , Derks G, Dijk DJ. Modelling changes in sleep timing and duration across the lifespan: changes in circadian rhythmicity or sleep homeostasis? Sleep Med Rev. 2016;28:96–107. 26545247 10.1016/j.smrv.2015.05.011

[23] Skeldon AC , Phillips AJ, Dijk DJ. The effects of self-selected light-dark cycles and social constraints on human sleep and circadian timing: a modeling approach. Sci Rep. 2017;7:45158. 28345624 10.1038/srep45158 PMC5366875

[24] Wulff K , Joyce E, Middleton B, Dijk DJ, Foster RG. The suitability of actigraphy, diary data, and urinary melatonin profiles for quantitative assessment of sleep disturbances in schizophrenia: a case report. Chronobiol Int. 2006;23(1–2):485–495. 16687321 10.1080/07420520500545987

[25] Klein T , Martens H, Dijk DJ, Kronauer RE, Seely EW, Czeisler CA. Circadian sleep regulation in the absence of light perception: chronic non-24-hour circadian rhythm sleep disorder in a blind man with a regular 24-hour sleep-wake schedule. Sleep. 1993;16(4):333–343. 8341894 10.1093/sleep/16.4.333

[26] MATLAB. 9.6.0.1135713 (R2019a) Update 3. Natick, MA: The MathWorks Inc., 2019.

[27] Fisher NI. Statistical Analysis of Circular Data. Cambridge: Cambridge University Press, 1993.

[28] Aldhous ME , Arendt J. Radioimmunoassay for 6-sulphatoxymelatonin in urine using an iodinated tracer. Ann Clin Biochem. 1988;25 (Pt 3):298–303. 3400987 10.1177/000456328802500319

[29] Arendt J , Bojkowski C, Franey C, Wright J, Marks V. Immunoassay of 6-hydroxymelatonin sulfate in human plasma and urine: abolition of the urinary 24-hour rhythm with atenolol. J Clin Endocrinol Metab. 1985;60(6):1166–1173. 3998065 10.1210/jcem-60-6-1166

[30] Borbély AA . A two process model of sleep regulation. Hum Neurobiol. 1982;1(3):195–204. 7185792

[31] Daan S , Beersma DGM, Borbely AA. Timing of human sleep: recovery´ process gated by a circadian pacemaker. Am J Physiol. 1984;246:R161–R183. 6696142 10.1152/ajpregu.1984.246.2.R161

[32] Kronauer RE , Forger DB, Jewett ME. Quantifying human circadian pacemaker response to brief, extended and repeated light stimuli over the phototopic range. J Biol Rhythms 1999;14:500–515. 10643747 10.1177/074873099129001073

[33] Klerman EB , Dijk DJ, Kronauer RE, Czeisler CA. Simulations of light effects on the human circadian pacemaker: implications for assessment of intrinsic period. Am J Physiol. 1996;270(1 Pt 2):R271–R282. 8769811 10.1152/ajpregu.1996.270.1.R271

[34] Meyer N , Kerz M, Folarin A, et al. Capturing rest-activity profiles in schizophrenia using wearable and mobile technologies: development, implementation, feasibility, and acceptability of a remote monitoring platform. JMIR Mhealth Uhealth. 2018;6(10):e188. 30377146 10.2196/mhealth.8292 PMC6234334

[35] Bei B , Manber R, Allen NB, Trinder J, Wiley JF. Too Long, too short, or too variable? sleep intraindividual variability and its associations with perceived sleep quality and mood in adolescents during naturalistically unconstrained sleep. Sleep 2016;40: zsw067. 10.1093/sleep/zsw067 28364491

[36] Bei B , Wiley JF, Trinder J, Manber R. Beyond the mean: a systematic review on the correlates of daily intraindividual variability of sleep/wake patterns. Sleep Med Rev. 2016;28:108–124. 26588182 10.1016/j.smrv.2015.06.003

[37] Kluge M , Himmerich H, Wehmeier PM, et al. Sleep propensity at daytime as assessed by Multiple Sleep Latency Tests (MSLT) in patients with schizophrenia increases with clozapine and olanzapine. Schizophr Res. 2012;135(1-3):123–127. 22257975 10.1016/j.schres.2011.12.017

[38] Reeve S , Sheaves B, Freeman D. Excessive sleepiness in patients with psychosis: an initial investigation. PLoS One. 2021;16(1):e0245301. 33449971 10.1371/journal.pone.0245301 PMC7810297

[39] Wright KP Jr , McHill AW, Birks BR, Griffin BR, Rusterholz T, Chinoy ED. Entrainment of the human circadian clock to the natural light-dark cycle. Curr Biol. 2013;23(16):1554–1558. 23910656 10.1016/j.cub.2013.06.039 PMC4020279

[40] Faulkner SM , Dijk DJ, Drake RJ, Bee PE. Adherence and acceptability of light therapies to improve sleep in intrinsic circadian rhythm sleep disorders and neuropsychiatric illness: a systematic review. Sleep Health. 2020;6(5):690–701. 32173374 10.1016/j.sleh.2020.01.014

[41] Moore-Ede MC , Sulzman FM, Fuller CA. The clocks that time us. Cambridge, Massachusetts and London, England: Harvard University Press, 1982.

[42] Faulkner SM , Bee PE, Meyer N, Dijk DJ, Drake RJ. Light therapies to improve sleep in intrinsic circadian rhythm sleep disorders and neuro-psychiatric illness: a systematic review and meta-analysis. Sleep Med Rev. 2019;46:108–123. 31108433 10.1016/j.smrv.2019.04.012

[43] Cajochen C . Alerting effects of light. Sleep Med Rev. 2007;11(6):453–464. 17936041 10.1016/j.smrv.2007.07.009

[44] Robillard R , Naismith SL, Rogers NL, et al. Delayed sleep phase in young people with unipolar or bipolar affective disorders. J Affect Disord. 2013;145(2):260–263. 22877966 10.1016/j.jad.2012.06.006

[45] Shochat T , Santhi N, Herer P, Dijk DJ, Skeldon AC. Sleepiness is a signal to go to bed: data and model simulations. Sleep. 2021:zsab123. 33991415 10.1093/sleep/zsab123 PMC8503825

[46] Ferrarelli F . Sleep Abnormalities in Schizophrenia: state of the Art and Next Steps. Am J Psychiatry. 2021; 178: 903– 913. 33726524 10.1176/appi.ajp.2020.20070968 PMC8446088

[47] Dijk DJ , Czeisler CA. Contribution of the circadian pacemaker and the sleep homeostat to sleep propensity, sleep structure, electroencephalographic slow waves, and sleep spindle activity in humans. J Neurosci. 1995;15(5 Pt 1):3526–3538. 7751928 10.1523/JNEUROSCI.15-05-03526.1995 PMC6578184

[48] Roenneberg T . Chronobiology: the human sleep project. Nature. 2013;498(7455):427–428. 23803826 10.1038/498427a

