# Supplementary material for: Extracting Circadian and Sleep Parameters from Longitudinal Data in Schizophrenia for the Design of Pragmatic Light Interventions
Source: Schizophr Bull. 2021 Nov 10;48(2):447–56. doi: 10.1093/schbul/sbab124 (PMC8886588; doi:10.1093/schbul/sbab124)
Supplement: sbab124_suppl_Supplementary_Material [file sbab124_suppl_supplementary_material.pdf]

Extracting sleep and circadian parameters from longitudinal data in schizophrenia for the design of pragmatic light interventions

Supplementary Material

Anne C Skeldon[1,2,*] Derk-Jan Dijk[2,3], Nicholas Meyer[4,5] Katharina Wulff [6,7]

[1] Department of Mathematics, Faculty of Engineering and Physical Sciences, University of Surrey, Guildford, GU2 7XH, UK
[2] UK Dementia Research Institute Care Research & Technology Centre, at Imperial College London and the University of Surrey, Guildford, UK
[3] Surrey Sleep Research Centre, Faculty of Health and Medical Sciences, University of Surrey, Guildford, GU2 7XP, UK
[4] Department of Psychosis Studies, Institute of Psychology, Psychiatry and Neuroscience, King's College London, UK
[5]Insomnia and Behavioural Sleep Medicine Clinic, Univeristy College London Hospitals NHS Foundation Trust, UK
[6] Departments of Radiation Sciences and Molecular Biology, Umeå Universitet, Sweden
[7] Wallenberg Centre for Molecular Medicine (WCMM), Umeå Universitet

[*] Coresponding author: a.skeldon@surrey.ac.uk

July 3, 2021

Further participant details

Living situations

At the time of the study, thirteen community-living patients lived on their own and 15 of the controls; one patient and 1 control each lived with a partner; one patient woman lived with a child and 1 control woman with two children; five single patients lived in shared accommodation (2 with parents, 2 in supported housing, 1 in hospital awaiting accommodation) and 4 single controls lived in shared flats (2-and-2).

Clinical status and medication

Activities of daily living and social activity by self-report and notes from mental health services was rated using the General Assessment of Function scale (GAF, Frances et al, 1994) and their score ranged from 34 to 70 (median 50) [1]. Behaviourally controlled, persistent hallucination or delusions were present in eight individuals. Regularly prescribed psychotropic medication to manage behavioural disturbances included antipsychotics only (n = 12), additional psychotropic medication to control extrapyramidal (antimuscarinics, n = 3) and seizure-inducing side effects of antipsychotics (anticonvulsants, n = 4). Depressive or anxiety symptoms were experienced by eight people at some point during their illness, two of these were treated with a selective serotonin inhibitor. Antipsychotic medication and dose (ranked by chlorpromazine equivalent), age, gender, length of illness, drug delivery mode and absence/presence of positive symptoms are detailed in Table **S1**.

For those with schizophrenia, illness duration ranged from 2 to 33 years (median 10 years). The patients were not high functioning but were average individuals with histories comparable to [2].

In [1], the presence of positive symptoms, age, length of illness, GAF and antipsychotic drug dose were not found to correlate with measures of sleep, rest-activity and melatonin, but differences in melatonin levels between individuals with and without positive symptoms were an effect of age. In our current study, we additionally found no correlation between measures of light and chlorpromazine equivalent (Spearman's rank: hours of bright light, $p = 0.97$; total light, $p = 0.74$; total log light $p = 0.50$).

Participant	Gender	Age (y)	Length of illness (y)	Antipsychotics	Anti-psychotic dose (mg)	CPZeq	Drug delivery mode	Positive symptoms (yes/no)
sz07	Female	39	4	Clozapine	500	1000	Oral/daily	Yes
sz06	Female	36	-	Clozapine,	350,	800	Oral/daily	Yes
			-	Amisulpride	200	800	Oral/daily	Yes
sz10	Male	46	27	Clozapine	350	700	Oral/daily	Yes
sz11	Male	56	33	Amisulpride	1000	500	Oral/daily	Yes
sz14	Male	26	5	Olanzapine	20	400	Oral/daily	Yes
sz03*	Male	27	6	Olanzapine	20	400	Oral/daily	Yes
sz02	Male	34	8	Olanzapine	20	400	Oral/daily	Yes
sz05	Male	32	11	Risperidone	50	300	Depot/2-weekly	Yes
sz15	Male	52	22	Risperidone	50	300	Depot/2-weekly	Yes
sz19	Male	43	12	Flupenthixol	50,	300	Depot/2-weekly	No
				Olanzapine	7.5	300	Oral/daily	No
sz20	Male	38	13	Zuclopenthixol	500	250	Depot/2-weekly	No
sz09	Male	47	10	Amisulpride	400	200	Oral/daily	No
sz16*	Female	49	10	Amisulpride	400	200	Oral/daily	No
sz12	Female	35	2	Risperidone	4	200	Oral/daily	Yes
sz04	Male	28	7	Olanzapine	10	200	Oral/daily	No
sz13	Male	45	7	Olanzapine	10	200	Oral/daily	No
sz21*	Male	28	4	Olanzapine	10	200	Oral/daily	No
sz01*	Female	35	12	Trifluoperazine	6	120	Oral/daily	No
sz17	Male	41	-	Olanzapine	5	100	Oral/daily	No
sz18	Male	41	23	Flupenthixol	50	75	Depot/4-weekly	No

Table S1: **Clinical descriptives** (adapted from supplementary Table DS1 in [1]). * Those highlighted in [1] as having non-24 h rhythms.

Monitoring of sleep-wake timing

In view of the functional disability associated with schizophrenia, to enhance data quality weekly home visits were made by the investigator. At these visits, actigraphy, light and diary entries were compared and any discrepancies discussed with the participant. This high level of supervision may not be practical in a clinical setting. However, in another recent study, we found surprisingly high rates of adherence with remote rest-activity monitoring in populations with chronic schizophrenia, some with high symptom burden of positive and negative symptoms [3]. We therefore believe that our approach is generalisable.

Monitoring of light exposure

Participants were instructed to keep the actiwatch unobscured and wear it on their non-dominant wrist. Nevertheless, there were occasions when participants were awake and lux values of 0 were recorded by the actiwatch. We therefore performed the analyses twice. First with the raw light data, as recorded. Second with values of 0 during wake replaced by imputed values. Imputation was done by using linear interpolation from the nearest non-zero values. This made very little difference to the outcome and made no change to the significance of reported associations. In general, in the mathematical model, missing light data has a greater impact on the simulation of sleep timing than on sleep duration and therefore affected values of intrinsic circadian period rather than wake drive. The absolute change in fitted intrinsic circadian period as calculated from imputed as compared with raw data varied from 0:00 h:m to 0.07 h:m with a median value of 0:02 h:m.

Method for finding melatonin circadian acrophase

Over a 48-h period once a week, 17 out of 20 people with schizophrenia and all 21 people in the control group completed repeated urine collection following a protocol suitable for people with schizophrenia [26]. These sequential measurements of aMT6s were subjected to a cosinor analysis implemented in MATLAB [28] to derive melatonin circadian acrophase. Only assessments in which a cosine was a significantly better fit than the mean ($p < 0.05$) were used in our analyses. Circadian acrophase was used as further validation of our modelling approach.

	Estimate (h:m)	Confidence interval (h:m)	Tstat	p	dF
	Sleep duration				
Intercept	7:30	(6:56 , 8:05)	25.52	$<$0.0001	1753
Group	2:24	(1:41 , 3:07)	6.57	$<$0.0001	1753
Gender	-0:01	(-0:48 , 0:45)	-0.03	0.9726	1753
	Sleep onset				
Intercept	01:38	(00:48 , 02:27)	3.88	0.0001	1753
Group	-0:30	(-1:31 , 0:32)	-0.92	0.3572	1753
Gender	-0:40	(-1:47 , 0:26)	-1.17	0.2398	1753
	Sleep offset				
Intercept	09:07	(08:10, 10:04)	18.81	$<$0.0001	1753
Group	2:07	(0:56, 3:18)	3.53	0.0004	1753
Gender	-0:37	(-1:54, 0:39)	-0.95	0.3447	1753
	Mid-sleep				
Intercept	05:25	(04:37 , 06:14)	13.09	$<$0.0001	1753
Group	0:36	(-0:25 , 1:37)	1.18	0.2382	1753
Gender	-0:47	(-1:52 , 0:19)	-1.39	0.1653	1753

Table S2: **Linear mixed effects model results** For 'Group' the reference category is controls. For 'Gender' the reference category is men.

Further statistical details and results

Linear mixed effect models were used to investigate the repeated measures of sleep duration, sleep onset and offset times. In all cases, fixed effects were participant group (control or schizophrenia) and gender. Participant was included as a random effect.

Results are summarised in Table **S2**. The effect of group on sleep measures is reported in the main text. We found no significant effect of gender.

Mathematical modelling

The model describes sleep and wake states as the firing of sleep-promoting and wake-promoting populations of neurons respectively. Switching between sleep and wake occurs as a result of homeostatic and circadian drives to sleep-promoting neurons. The homeostatic sleep drive increases with time awake, as the physiological pressure for sleep increases. The circadian drive models oscillatory input from the master circadian clock. During the biological day the circadian clock promotes wakefulness whereas over the biological night it promotes sleep [4–6].

In addition, the model captures the impact of light exposure and endogenous circadian factors on sleep timing by so-called process L [7, 8]. Light-dark information reaches the biological clock via the eye and consists of the solar light-dark cycle and electric light. Light exposure is under behavioural control, i.e. during the day our behaviour determines our exposure to daylight, how brightly we light our homes, and whether we turn the lights off when we go to bed. The timing and synchronisation of the biological clock and sleep-wake cycle are directly related to the 'strength' of the light-dark cycle and the extent to which the intrinsic circadian period deviates from 24 h. 'Strength' is the relative balance between light exposure in the morning, which tends to shift sleep earlier, and the light exposure in the evening, which tends to delay sleep. The more an individual's intrinsic period deviates from 24 h the stronger the light-dark cycle needed to maintain rhythmicity. When the period is much longer than 24 h or the contrast between light and dark too small, the clock and sleep will no longer be in synchrony with the 24-h day and sleep will drift later and later on each successive day.

Equations

As in [4, 5], the interaction between sleep promoting and wake promoting neurons was described by equations for their mean electric potential, V_v and V_m, respectively,

$$\tau \frac{dV_v}{dt} = -V_v - \nu_{vm} Q_m + D_v, \tag{1}$$

$$\tau \frac{dV_m}{dt} = -V_m - \nu_{mv} Q_v + D_m. \tag{2}$$

Here,

$$Q_{v,m} = \frac{Q_{\max}}{1 + \exp[-(V_{v,m} - \theta)/\sigma]}, \tag{3}$$

describes the sigmoidal relationship between the potential and the firing rate of the neurons, $Q_{v,m}$. The parameter $Q_{\max}$ is the maximum possible firing rate; θ is the value of the potential $V_{v,m}$ at $Q_{v,m} = Q_{\max}/2$ and σ determines the width of the sigmoid.

The parameter τ gives the typical timescale of the neuronal process and the parameters $\nu_{vm,mv}$ weight the input from population m to v and v to m respectively.

The $D_{v,m}$ are 'drives'. The drive to the wake promoting neurons, D_m was fixed. The drive to the sleep promoting neurons, D_v, consisted of homeostatic and circadian components,

$$D_v = A_v - \nu_{vc} C(t) + \nu_{vh} H(t), \tag{4}$$

where $H(t)$ describes the homeostatic sleep pressure and $C(t)$ represents the circadian wake promoting rhythm. As discussed further below, $C(t)$ is approximately sinusoidal with amplitude close to one, so ν_{vc} gives the amplitude of the circadian wake propensity rhythm.

The term A_v is a background excitatory input to the sleep promoting neurons.

The homeostatic process is given by

$$\chi \frac{dH}{dt} = -H + \mu Q_m. \tag{5}$$

Here, H/χ is the rate of removal of somnogenic chemical and $\mu Q_m/\chi$ is the rate of production.

The circadian rhythm $C(t)$ was modelled by a forced van der Pol oscillator [8], where the forcing represents the light intensity dependent signal to the suprachiasmatic nucleus from photoreceptors in the eye. The model was originally constructed to accurately replicate human phase response data [8]. Specifically,

$$\kappa \frac{dx}{dt} = \gamma \left(x - \frac{4x^3}{3} \right) - y \left(\left(\frac{24}{f \tau_c} \right)^2 + kB \right), \tag{6}$$

$$\kappa \frac{dy}{dt} = x + B, \tag{7}$$

$$\frac{dn}{dt} = \lambda \left(\alpha_0 \left(\frac{\tilde{I}}{I_0} \right)^p (1-n) - \beta n \right), \tag{8}$$

where

$$B = \alpha_0 G (1-n)(1-bx)(1-by) \left(\frac{\tilde{I}}{I_0} \right)^p, \tag{9}$$

Here, x and y are the variables for the van der Pol oscillator and n is the fraction of activated photoreceptors. The term B describes the forcing that occurs as a result of the action of light of intensity $\tilde{I}$ on photoreceptors. The magnitude of B is dependent on phase, as modelled by the term $(1-bx)(1-by)$ in equation (9). The rate of saturation of photoreceptors is given by $\lambda \alpha_0 \left(\frac{\tilde{I}}{I_0} \right)^p$ and the rate for decay is given by $\lambda \beta$, where the values of $\lambda, \alpha_0, I_0, p$ and β have been determined from experimental data [7]. The parameter τ_c is the intrinsic period, γ is the stiffness of the oscillator.

Note that the intrinsic period of the van der Pol oscillator was dependent on light intensity since for diurnal species intrinsic period is shorter for higher light intensities (Aschoff's rule). This dependence of the intrinsic period on light intensity is controlled by the parameter k in equation (6).

When light intensity $I(t)$ is provided by measurements in the field, we make the assumption that the relationship between light intensity $\tilde{I}$ incident on photoreceptors in the eye and measured light is $\tilde{I} = I(t)$. In this case, information on sleep timing is implicitly given within the light data since lights tend to be turned off during sleep.

When using the model in predictive mode, we make no assumption that light is turned off at night and light is 'available' at all times of day. In these cases, $\tilde{I}$ is modelled as

$$\tilde{I} = \mathcal{H}(Q_m - Q_{th})I(t), \tag{10}$$

where $\mathcal{H}$ is the Heaviside function. This means that light impinging on photoreceptors is zero if the firing rate of wake promoting neurons, Q_m, is greater than a threshold value $Q_{\rm th}$ i.e. light is turned off during model-predicted sleep. See Fig. **S1**(a).

As in [5], the circadian wake propensity rhythm $C(t)$, was modelled as

$$C(t) = \frac{1}{2}\left(1 + 0.80y - 0.47x\right). \tag{11}$$

Sleep/wake regulation parameters					
$Q_{\rm max}$	100 s^{-1}	θ	10 mV	σ	3 mV
ν_{vm}	2.1 mVs	ν_{mv}	1.8 mVs	ν_{vc}	3.84 mV
ν_{vh}	1 mVnM^{-1}	A_m	1.3 mV	A_v	fitted
τ	10 s			χ	$45 \times 60 \times 60$ s
μ	4.41 nMs	$Q_{\rm th}$	1 s^{-1}		
**Circadian parameters**					
κ	$\frac{12}{\pi} \times 60 \times 60$ s	γ	0.23	f	0.99669
τ_c	fitted	k	0.55	α_0	0.16
G	19.9				
I_0	9500 lux	β	0.013	b	0.4
p	0.6				

Table S3: **Parameter values**

All the parameters values used are listed in Table **S3**. The sleep/wake regulation and circadian parameters are the same as those used in the original version of the model [4]. Equations were integrated using the stiff solver ODE15s in MATLAB [9]. A typical solution for equations (1)-(11) is shown in Fig. **S1**.

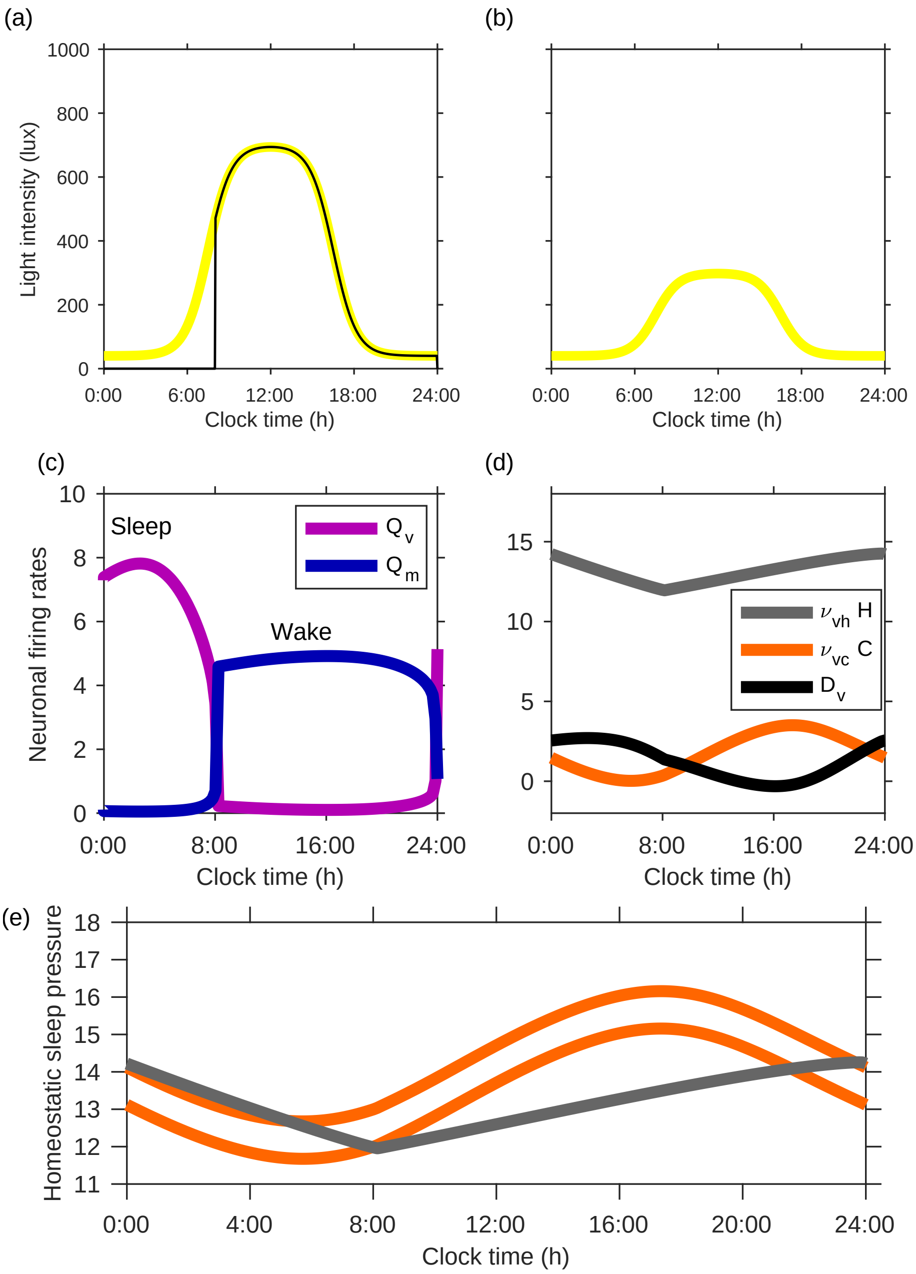

Figure S1: **Typical simulation output.** (a) and (b) typical light profiles (yellow) given by equation (12) for l_1 = 700, 300 respectively; l_2 = 40 in both cases. All other parameters are given in Table **S3**. In (a), the thin black line shows the light profile gated by the sleep-wake cycle for the particular case shown in (c)-(e). (c) The firing rates for the wake promoting and sleep promoting neurons, Q_m and Q_v respectively. (d) The homeostatic sleep drive, $\nu_{vh}H$ (dark grey) and the circadian wake propensity $\nu_{vc}C$ (orange). The difference between these two components gives the sleep drive D_v (black). (e) The numerical data re-plotted in the form of the two process model. The upper and lower thresholds are modulated by the circadian wake propensity rhythm. For (c)-(e), the light profile illustrated in (a) was used; μ = 4.20, ν_{vc} = 3.37, τ_c =24.2. All other parameter values are given in Table **S3**. This figure is reproduced from the Supplementary Material for [5].

Modelling changes in sleep duration and timing

In equations (1) - (5), sleep duration is determined by three separate groups of parameters. First, parameters which relate to the rate of increase of homeostatic sleep pressure (μ and χ). Second, by parameters which relate to the thresholds at which switching between wake and sleep occur (A_v and A_m) and finally, by ν_{vh} which determines the sensitivity to homeostatic sleep pressure. We note that there is a scaling between ν_{vh} and μ which means that mathematically these two parameters are equivalent: one of them could be scaled from the equations. Here, as in previous publications we retain both.

Previously, we have modelled changes in sleep duration across the lifespan by changing the parameter μ [10]. However, studies of slow wave sleep provide no evidence that those with schizophrenia have a higher sleep need [11]. Hence here we considered the parameters that govern wake propensity, namely A_v and A_m. Wake propensity could be reduced by lowering the baseline input to wake promoting neurons (A_v). However, paradoxically, with the form we have taken for the rate of production of somnogenic chemicals in equation (5), reducing the drive for wake results in a decrease in sleep duration. This is because of a 'use dependent' effect: reducing the drive for wake also reduces the firing rate of the wake promoting neurons and thus the build up of homeostatic sleep pressure during wake. Hence, here we have modelled reduced wake propensity by reducing the mean level of the input to sleep promoting neurons, A_v in equation (4). This is equivalent to lowering both thresholds of the wake propensity rhythm in the two process model [12–14] and also Fig. **S2**.

Parameters that control sleep timing include the intrinsic circadian period τ_c in equation (7) and the circadian amplitude, ν_{vc}. Here, since there are known individual differences in intrinsic circadian period, [15], we elected to vary intrinsic circadian period, see Fig. **S2**.

Parameter fitting

For each individual, we selected the value of A_v that matched mean sleep duration in the model to mean observed sleep duration. Since there is a monotonic relation between sleep duration and A_v, see Fig. **S2**, this is straightforward.

Next, for each individual, we fed the raw light data into the mathematical model and selected the intrinsic period so that mean mid-sleep time in the model output matched mean observed mid-sleep time.

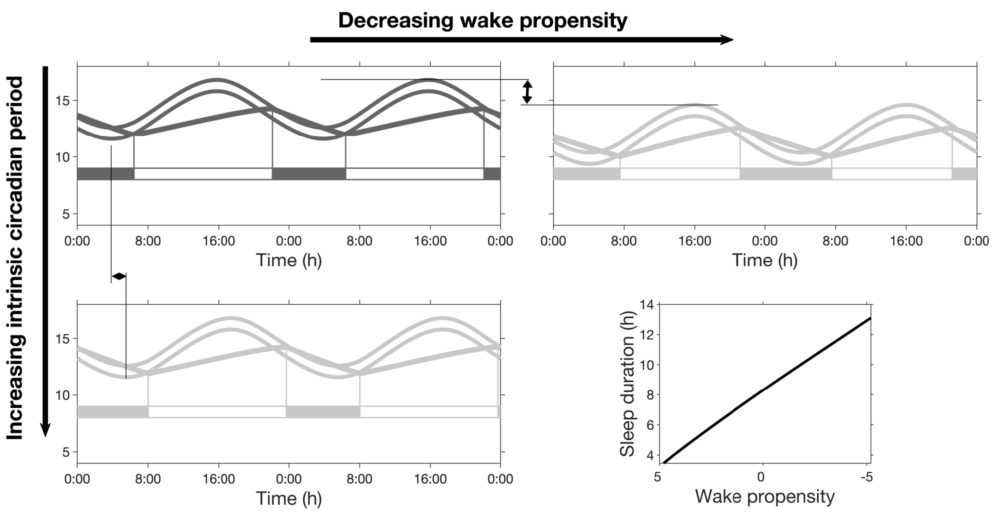

Figure S2: **Effect of reducing wake propensity and increasing intrinsic period**Parameter values: top left, τ_c = 24.2 h, A_v = -10.2; top right, τ_c = 24.2 h, A_v = -8.0; bottom left, τ_c = 24.5 h, A_v = -10.2. Bottom right, wake propensity as measured by deviation from the default value for A_v, with negative numbers indicating decreased wake propensity i.e. wake propensity = A_v + 10.2. Note, here for consistency with the upper panels, the horizontal axis is scaled so that lower values of wake propensity, hence longer sleep duration, is to the right.

Performing the fitting sequentially, as described, is possible because changing intrinsic period has minimal effect on sleep duration. It is computationally more efficient that simultaneously fitting for both parameters.

We investigated whether there were gender differences in the distributions of fitted parameters by using the Wilcoxon rank-sum test. For fitted wake propensity, there was no significant difference between men and women (p = 0.409). In line with previous observations [15], fitted intrinsic circadian period was on average slightly longer in men than in women, but did not reach significance in our sample (p = 0.051).

Modelling light interventions

Motivated by the shape of real world light profiles [16, 17] and as introduced in our previous work [5], we used the function

$$I(t) = l_2 + \frac{(l_1^i - l_2)}{2} \left\{ \tanh\left(c(\mathrm{mod}(t, 24 \times 60 \times 60) - s_1)\right) - \tanh\left(c(\mathrm{mod}(t, 24 \times 60 \times 60) - s_2)\right) \right\}. \tag{12}$$

This ramps up to a maximum level l_1^i during core day light hours on day i and reduces to a baseline of l_2 during the evening, where the transition from l_2 to l_1 occurs around $t = s_1$ and from l_1 to l_2 around $t = s_2$. The speed of the transition is determined by the parameter c.

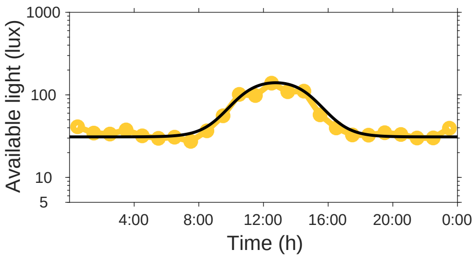

Figure S3: **Mean light profile for participant sz01 in the winter.** In yellow are the hourly average values. In black is the profile given by equation (12) for l_1 = 170 lux, l_2 = 30 lux, s_1 = 10:30 h, s_2 = 15:00 h.

For appropriate parameter choices, the profile given by equation (12) gives a good match to the mean hourly light intensity profile observed in the winter by participant sz01 who exhibited non-24 h rhythms, see Fig. **S3**.

In order to systematically investigate the effect of different light profiles, the values for the 'evening light' l_2 and the 'day light' l_2 were varied over a range of relevant values. Four different combinations of the physiological parameters were used to demonstrate the impact of intrinsic period τ_c and wake propensity A_v on the regions of entrainment. The results are shown in Fig. **S4**. As one might expect, these show that increasing intrinsic circadian period makes it harder to entrain to the 24 h day. Thus those with a long intrinsic period would need higher levels of light during the day and/or lower levels of light in the evening to achieve a desired spontaneous wake time than those with an intrinsic circadian period closer to 24 h. Decreased wake propensity can either make it easier or harder, depending on the intrinsic period. Note that the longer sleep duration means that a reduced wake propensity makes it harder to get up early in the morning.

The interaction between wake propensity and intrinsic period means that without the mathematical model it is hard to predict the outcome of a change in available light.

We note that the overall shape of the entrainment regions is consistent with the way that the Arnold entrainment tongues [5] shift as a function of evening light and day light.

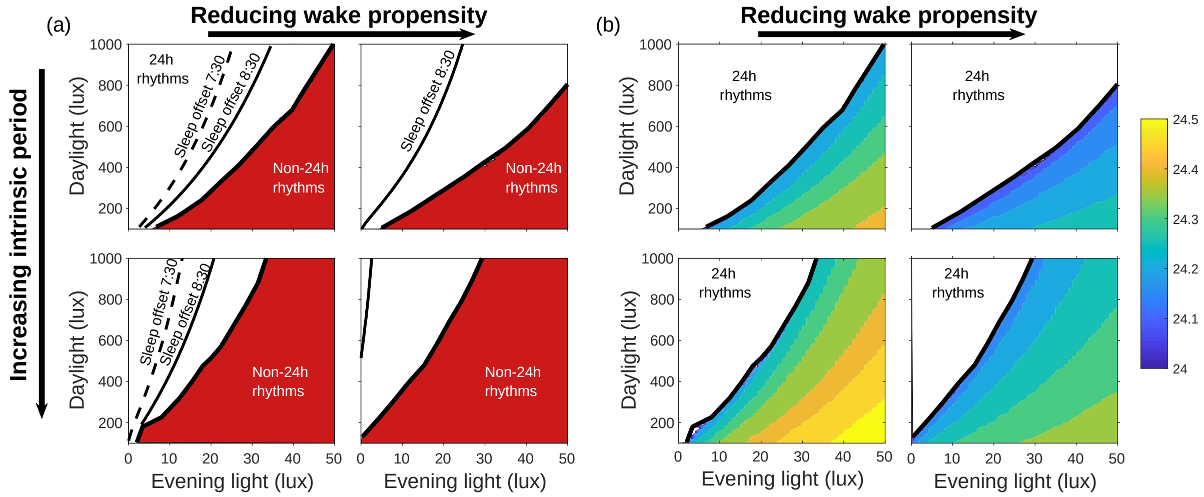

Figure S4: **Regions of entrainment** (a) Entrainment regions with example sleep offset times for four different sets of physiological parameters. (b) The same entrainment regions, but with the region where non-24 h rhythms occur shaded according to the mean period of the sleep-wake cycle. Parameter values: top left, τ_c = 24.2 h, A_v = -10.2, top right, τ_c = 24.2 h, A_v = -6, bottom left, τ_c = 24.3 h, $A_v = -10.2$, bottom right, τ_c = 24.3 h, $A_v = -6$. A_v is the 'drive for sleep'. So increasing values of A_v correspond to reduced wake propensity.

References

1. Wulff, K., Dijk, D.-J., Middleton, B., Foster, R. G. & Joyce, E. M. Sleep and circadian rhythm disruption in schizophrenia. *Brit. J. Psychiat.* **200,** 308–316 (2012).
2. Jepson, J. A. My Stages of Recovery. *Schizophrenia Bull.* **43,** 3–5 (Mar. 2014).
3. Meyer, N. *et al.* Capturing Rest-Activity Profiles in Schizophrenia Using Wearable and Mobile Technologies: Development, Implementation, Feasibility, and Acceptability of a Remote Monitoring Platform. *JMIR Mhealth Uhealth* **6,** e188 (2018).
4. Phillips, A. J. K., Chen, P. Y. & Robinson, P. A. Probing the Mechanisms of Chronotype Using Quantitative Modeling. *J. Biol. Rhythms* **25,** 217–227 (2010).
5. Skeldon, A. C., Phillips, A. J. K. & Dijk, D.-J. The effects of self-selected light-dark cycles and social constraints on human sleep and circadian timing: a modeling approach. *Sci. Rep.* **7,** 45158 (2017).
6. Phillips, A. J. K. & Robinson, P. A. A Quantitative Model of Sleep-Wake Dynamics Based on the Physiology of the Brainstem Ascending Arousal System. *J. Biol. Rhythms* **22,** 167–179 (2007).
7. Kronauer, R. E., Forger, D. B. & Jewett, M. E. Quantifying human circadian pacemaker response to brief, extended and repeated light stimuli over the phototopic range. *J. Biol. Rhythms* **14,** 500–515 (1999).
8. Forger, D. B., Jewett, M. E. & Kronauer, R. E. A simpler model of the human circadian pacemaker. *J. Biol. Rhythms* **14,** 533–537 (1999).
9. MATLAB. *9.6.0.1135713 (R2019a) Update 3* (The MathWorks Inc., Natick, Massachusetts, 2019).
10. Skeldon, A. C., Derks, G. & Dijk, D.-J. Modelling changes in sleep timing and duration across the lifespan: Changes in circadian rhythmicity or sleep homeostasis? *Sleep Med. Rev.* **28,** 96–107 (2016).
11. Winsky-Sommerer, R. *et al.* Disturbances of sleep quality, timing and structure and their relationship with other neuropsychiatric symptoms in Alzheimer's disease and schizophrenia: Insights from studies in patient populations and animal models. *Neurosci. Biobehav. R.* **97,** 112–137 (2019).
12. Borbély, A. A. A two process model of sleep regulation. *Hum. Neurobiol.* **1,** 195–204 (1982).
13. Daan, S., Beersma, D. G. M. & Borbély, A. A. Timing of human sleep: Recovery process gated by a circadian pacemaker. *Am. J. Physiol.* **246,** R161–R183 (1984).
14. Skeldon, A. C., Dijk, D.-J. & Derks, G. Mathematical Models for Sleep-Wake Dynamics: Comparison of the Two-Process Model and a Mutual Inhibition Neuronal Model. *PLoS ONE* **9,** 1–16 (2014).
15. Duffy, J. F. *et al.* Sex difference in the near-24-hour intrinsic period of the human circadian timing system. *P Natl Acad. Sci. USA* **108,** 15602–15608 (2011).
16. Thorne, H. C., Jones, K. H., Peters, S. P., Archer, S. N. & Dijk, D.-J. Daily and Seasonal Variation in the Spectral Composition of Light Exposure in Humans. *Chronobiol. Int.* **26,** 854–866 (2009).
17. Wright, K. P. *et al.* Entrainment of the Human Circadian Clock to the Natural Light-Dark Cycle. *Curr. Biol.* **23,** 1554–1558 (2013).
